# Long-term clinical course and prognosis of vaccine-related persistent itching nodules (1997–2019): An observational study

**DOI:** 10.1016/j.jvacx.2022.100163

**Published:** 2022-04-29

**Authors:** Anette Gente Lidholm, Annica Inerot, Martin Gillstedt, Elisabet Bergfors, Birger Trollfors

**Affiliations:** a Department of Dermatology and Venereology, Institute of Clinical Sciences, Sahlgrenska Academy, University of Gothenburg, Gothenburg, Sweden; b Department of Dermatology and Venereology, Region Västra Götaland, Sahlgrenska University Hospital, Gothenburg, Sweden; c General Practice / Family Medicine, School of Public Health and Community Medicine, Institute of Medicine, Sahlgrenska Academy, University of Gothenburg, Gothenburg, Sweden; d Department of Paediatrics, Sahlgrenska Academy, University of Gothenburg, Gothenburg, Sweden

**Keywords:** Childhood vaccine, Adverse event, Aluminium, Aluminium allergy, Itching nodules, Granulomas, aP, Acellular pertussis toxoid vaccine, ASIT, Allergen-specific immunotherapy, DTaP, Diphtheria-tetanus-acellular pertussis vaccine, DT, Diphtheria-tetanusvaccine, SSI, Statens Serum Institut, HPV, Human papillomavirus, TBE, Tick-borne encephalitis

## Abstract

**Background:**

Vaccines adsorbed to aluminium can induce long-lasting intensely itching subcutaneous nodules (granulomas) at the injection site as well as contact allergy to aluminium. In clinical trials of a new acellular pertussis vaccine performed in the 1990s (Gothenburg, Sweden) with 76 000 participants, itching nodules were reported in 745 children. A positive patch test to aluminium was verified in 77% of the tested children with itchy nodules.

**Aim:**

To describe the long-term clinical course and prognosis of vaccine-related itching nodules caused by aluminium-containing pediatric vaccines and to estimate the risk for new symptoms after future vaccination with aluminium-containing vaccines.

**Methods:**

745 children with vaccine-related itching nodules were followed by regular interviews/questionnaires for more than 20 years. 723 of them received a booster dose of diphtheria/tetanus vaccine either with or without aluminium adjuvant during the follow-up time.

**Results:**

Most study participants (86%) reported a full recovery from their itching nodules after a median duration of 6.6 years. Only a few of the diphtheria/tetanus-booster-vaccinated children (3%) reported mild transient itching and swelling at the new injection site.

**Conclusion:**

Vaccine-induced itching granulomas caused by an aluminium-adsorbed acellular pertussis toxoid vaccine seem to disappear over time. Future vaccinations with aluminium-adsorbed vaccines can be performed with little risk for new itching nodules later in life.

## Highlights

• Vaccine-induced itching nodules almost always resolve over time.
• The symptoms may last up to six years though diminishing with time.
• Contact allergy to aluminium is not a cause to refrain from further vaccination.
• Vaccination can continue once the nodule has vanished and the itching has resolved.

## Introduction

Vaccines adsorbed to aluminium-based adjuvants can induce long-lasting, intensely itching subcutaneous nodules (granulomas) at the injection site [1], [2], [3] as well as contact allergy to aluminium [1], [4], [5], [6]. Aluminium containing adjuvants, mostly aluminium hydroxide, are used in vaccines targeting diphtheria, tetanus, pertussis, hepatitis A and B, human papillomavirus and tick-borne encephalitis as well as in pneumococcal and meningococcal conjugate vaccines. Itching granulomas are described after use of most of these vaccines [7], [8], [9] and also after treatment with aluminium-adsorbed allergen-specific immunotherapy (ASIT) [10], [11], [12].

Both itching granulomas and contact allergy to aluminium were considered uncommon and only described in case reports [2], [13], [14] until 2003, when persistent itching nodules were reported in 745 of 76 000 children (0.98%) [1], [15] in clinical trials of an acellular pertussis toxoid vaccine in Gothenburg, Sweden [16], [17].

Contact allergy to aluminium was verified with an epicutaneous test in 77 % of the children with vaccination granulomas in the Gothenburg trials [1]. Other typical findings were a remarkably long delay between vaccination and onset of symptoms (median three months), intense itching of the skin overlying the nodule, local eczema, hypertrichosis and discolouration in the itching area. Exacerbation of symptoms during intercurrent infections was common as well as very long symptom duration. When the course and prognosis of persistent itching nodules were first reported in 2003, 75% of the children still experienced ongoing symptoms after a median duration of 4 years [1].

In 2013 we described that the observed aluminium allergy could be reversible after many years [15].

The aims of the present study were to describe (I) the long-term clinical course and prognosis of vaccine-related itching nodules, and (II) the outcome after further vaccination with aluminium-adsorbed vaccines by following the afflicted children regularly for a median time of 15 years. This has to our knowledge not been studied before and will therefore contribute to increased knowledge of the long-time prognosis for persistent itching vaccination granulomas.

## Patients and methods

**Fig. 1:**
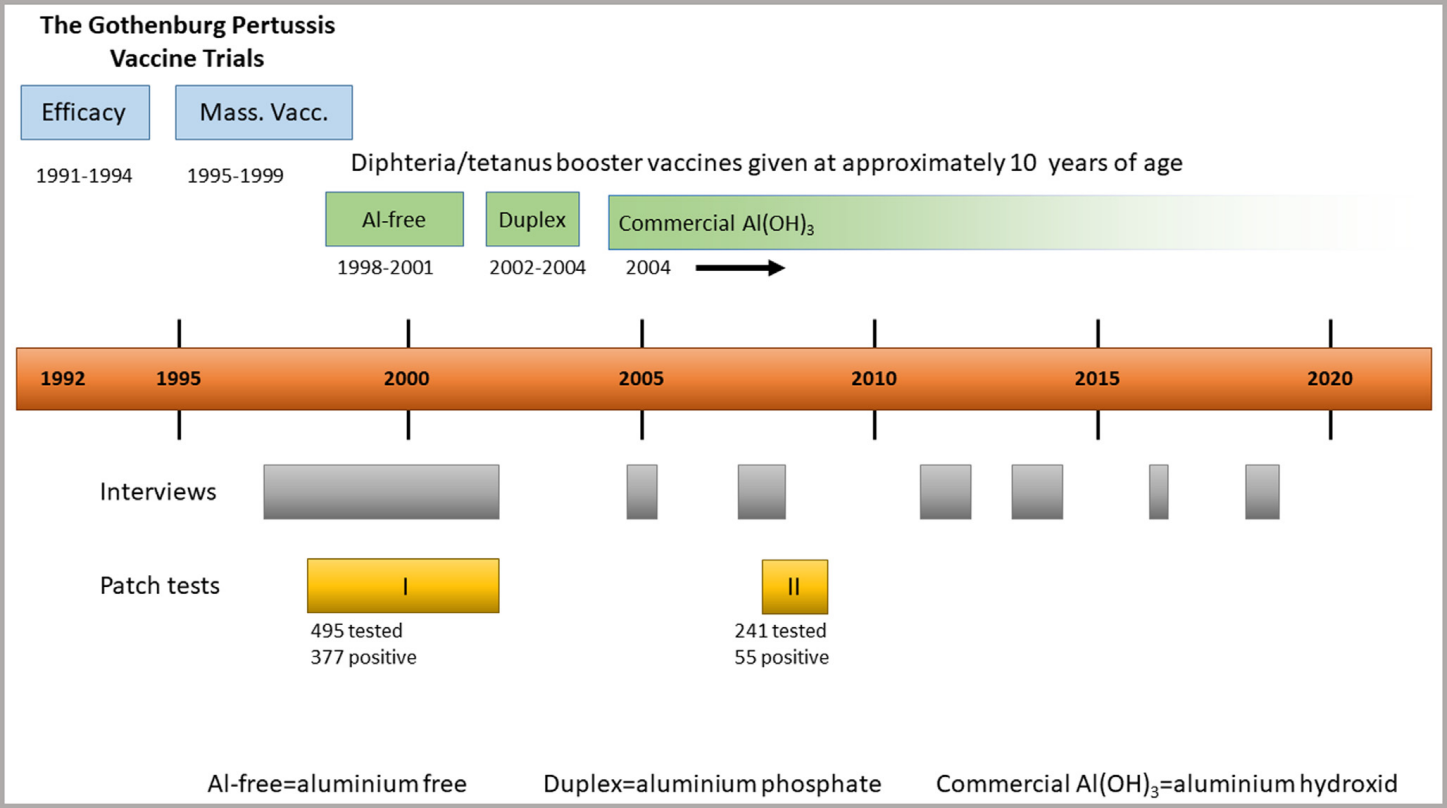
Schematic time-line (1991–2019) and overview of the follow-up study of 745 children with persistent itching nodules in the Gothenburg Pertussis Vaccine Trials: The trials, the DT-booster vaccinations, the interviews concerning the clinical course and the patch test studies.

A timeline overviewing the vaccinations, interviews and patch testings related to the vaccine trials is shown in Fig. 1.

### Patients

All individuals included in this study (n = 745; 455 girls, 290 boys; born in 1982–1999), participated in clinical trials in Gothenburg, Sweden [1], [15] and presented with adverse events consisting of persistent subcutaneous itching nodules at the injection site after vaccination with a new acellular pertussis toxoid vaccine (aP). The aP was either given alone or in combination with a diphtheria-tetanus toxoid vaccine (DTaP). Both vaccines contained aluminium hydroxide corresponding to 0.5 mg aluminium/dose and were produced by Statens Serum Institut (SSI, Copenhagen, Denmark).

About 76 000 children participated in the trials, most of them in a mass vaccination project initiated in 1995 [16] and some in an earlier double-blind efficacy trial [17] and a smaller immunogenicity study [18]. For detailed information on the vaccination schedules, see Bergfors et al [1]. Persistent itching nodule were defined as one or more subcutaneous nodules at the vaccination site and more or less intense pruritus in the overlying skin for at least six months.

## Methods

### Interviews and questionnaires

In 1997, when the reported number of children with itching nodules on their thigh or upper arm rapidly increased, a clinical follow-up was initiated within the Gothenburg Mass Vaccination Project. It comprised physical examinations of the child by one of the study physicians and repeated structured interviews by telephone or questionnaire performed by the trial staff. The symptoms were then followed by telephone contacts every month to every sixth month, depending on the frequency and severity of the itching and the condition of the skin. The symptoms could vary from – at worst – intense itching every day until the skin was bleeding and night sleep was disturbed to – later in the course - periods of mild itching, often preceded by a common cold, alternating with symptomless periods for weeks or months.

Later, interviews were replaced by written questionnaires sent out in intervals of 1 to 3 years. The same questions was asked in every contact throughout the study period: Does the child still have itching at the old vaccination site? Symptoms were graded in 4 stages: Unchanged; improved; nearly recovered; recovered. “Recovered” was defined as both nodules and itching having ceased for at least 6 months, see Table 1 for complete definition [1]. Later questions were: At about what time did the itching cease? In unclear cases, the end of symptoms was approximated. Has the child received any other aluminium-containing vaccines, and if so: which? Did the child get another itching nodule at the new vaccination site? A local warm red swelling occurring a few days after vaccination was interpreted as an unspecific mild local reaction and not as a vaccine-induced long-lasting itching nodule. Information regarding exposure to aluminium-containing products (antiperspirants, sunscreen protectors) was also inquired. The follow-up reported here continued through 2019.

**Table 1:** Definitions of symptoms for children with persistent itching nodules in the Gothenburg Pertussis Vaccine Trials [19]. “Unchanged symptoms” relates to the intensity of symptoms in the beginning of the course when they were at worst.

State of symptoms	Nodules	Itching
Unchanged symptoms	Unchanged	More or less continuous
Improved	Intermittent or diminished	Free periods for some weeks
Nearly recovered	Vanished or intermittent	Free periods for some months
Recovered	Vanished for ≥ 6 months	None during ≥ 6 months

The last questionnaires to all 745 children were sent out in 2011–2012, followed by telephone interviews in 2013–2014 with those who did not answer. The frequency of answers in the interviews was approximately 80% during 2011–2014. From there on, the interviews/questionnaires focused on two groups of study participants: those who reported continued itching at the last contact and those with no further contact since 2008, regardless of symptoms (usually moved abroad). When the children turned 18, they were interviewed themselves instead of their parents. These interviews were performed by the authors and by nurses and a medical laboratory technician, most of whom had taken part in the clinical trials from the beginning.

Beyond regular interviews or questionnaires, spontaneous contact for information and advice was taken by the parents or the participants themselves throughout the whole study period. In each spontaneous contact the same questions were asked as in the structured interviews.

From 2000 all child health centers, school nurses, paediatricians and dermatologists in the area were informed about itching nodules and their relation to the vaccination and asked to refer new cases to the study [1]. All data, including vaccination history, were documented in individual medical records.

As part of the follow-up study, the childrens caregivers were informed of the association between the aluminium content of the vaccine and the itching nodules and that they were benign but could be long-lasting.

### Patch testing for aluminium allergy

From 1998 occasional children had been patch tested with aluminium for detecting delayed hypersensitivity. Two years later, all known children with itching nodules were offered the same test (Patch test I). In total 495 children performed the test, 377 with positive results [1], [15]. In patch test II (2007–2008) 241 children who tested positive in 1998–2002 were retested. Of these 55 still tested positive [15]. The aluminium compound used in patch test I and II was aluminium chloride hexahydrate 2% in petrolatum and an empty FinnChamber®. For details on the testing see ref [1], [15].

On both patch test occasions, each individual was interviewed and examined at the original injection site. Further physical examination has not been done within the present study.

### Future vaccinations

According to the Swedish Childhood Vaccination Program, the next-coming planned vaccination for the study children born in 1988 or later was a booster dose of DT vaccine given as part of the School Health Care program at 10 years of age. This vaccine, Duplex® (SBL, Stockholm, Sweden), was adsorbed to aluminium phosphate.

To minimise the risk of developing new itching nodules caused by an aluminium adjuvant, the children in turn for booster vaccination received a licenced aluminium-free DT vaccine (SSI, Copenhagen, Denmark) within the Gothenburg Mass Vaccination Project from 1998 to 2002, when the aluminium-free vaccine was no longer produced. By then a new DT-booster vaccine, diTeBooster®, from the same manufacturer and containing the same aluminium hydroxide adjuvant as the pertussis vaccines in the Gothenburg Trials, had then been introduced in the School Health Care program. In this situation, Duplex® was, after all, considered a better choice to provide to the afflicted children since it had been used in Sweden for decades without any reports of persistent itching nodules. From 2005, when the production of this vaccine also ceased, the remaining study children received their DT-booster in school with commercial vaccines (Table 2).

**Table 2:** Diphtheria/tetanus-booster (DT-booster) vaccines given to the study children with itching granulomas at approximately 10 years of age.

Period	Vaccine	Aluminium content per dose	Manufacturer	Vaccination administered by
1997–1999	Duplex®	Aluminium phosphate 1.25 mg	SBL, Stockholm, Sweden	School Health Care
1999–2002	Diphtheria-Tetanus vaccine without aluminium	0	SSI, Copenhagen, Denmark	Gothenburg Pertussis Vaccine Trial
2003–2005	Duplex®	See above		Gothenburg Pertussis Vaccine Trial
2005	diTeBooster®	Aluminium hydroxide hydrate 0.5 mg	SSI, Copenhagen, Denmark	School Health Care

Since the 1990 s, a new aluminium-absorbed vaccine has been included in the Swedish Childhood Vaccination Program, namely human papillomavirus (HPV) for girls given by the School Health Care program at 10–12 years of age. Other aluminium-containing vaccines frequently used in Sweden during the last decades are those against hepatitis A and B and tick-borne encephalitis (TBE). All these vaccinations were inquired about in the last interviews/questionnaires.

## Ethical considerations

The studies were approved by the Regional Ethical Review Board of Gothenburg University in 2007 (reference number 385–07) and 2011 (623–11). All parents, adolescents and young adults received oral and written information about the study, and all parents and/or young adults gave written consent to participate.

## Statistical analysis

This is a descriptive study with no comparison group.

## Results

### Symptom duration

**Fig. 2:**
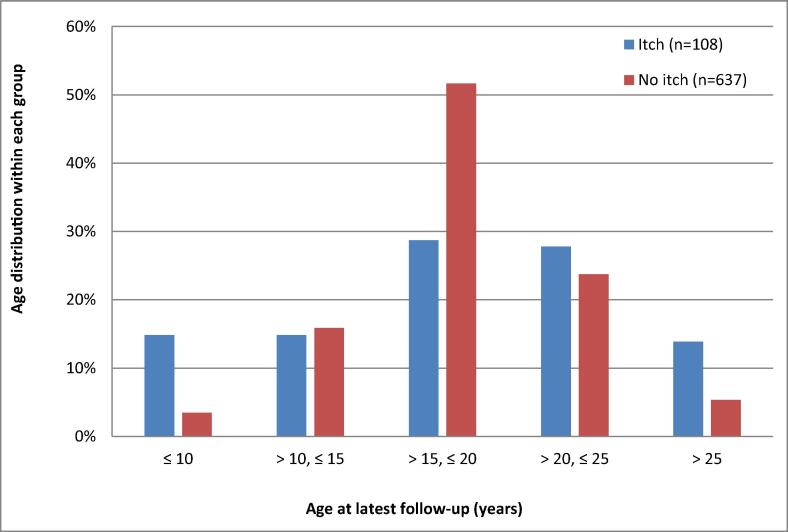
Study participants with vaccine-related itching nodules: Age at last contact.

The individual follow-up time from the start of symptoms until the last contact for each of the 745 children/adolescents with itching nodules varied between 0.1 and 25.2 years (median 15.0 years). For details of age at last contact and status of symptoms, see Fig. 2.

**Fig. 3:**
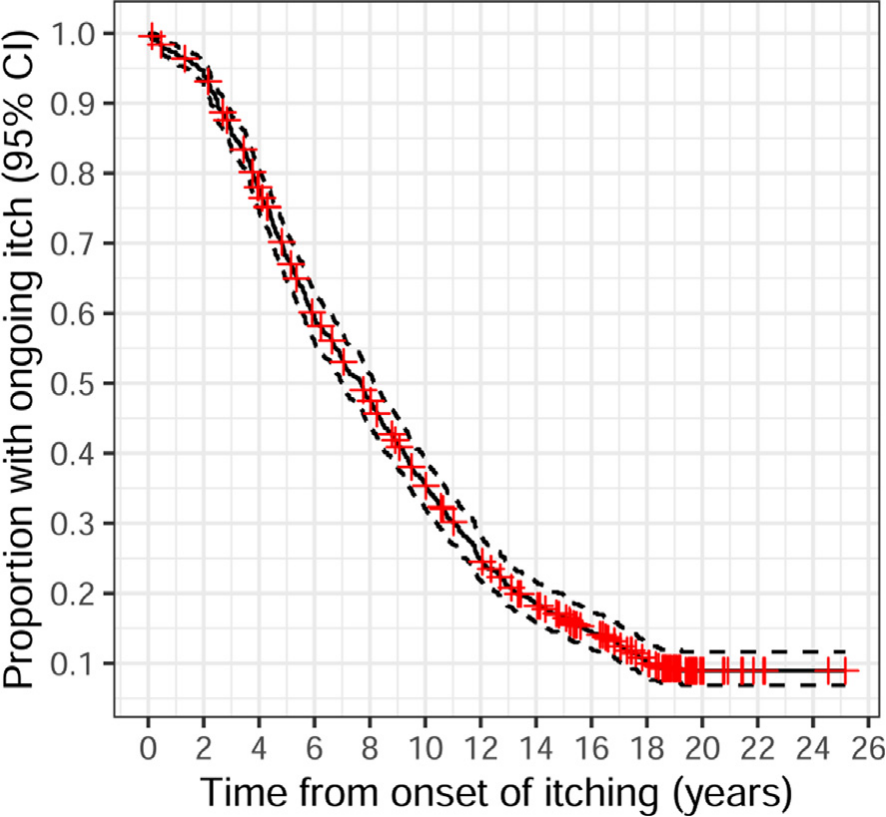
Kaplan-Meier plot of the proportion of children with ongoing symptoms. Time zero is defined for each of the 745 individuals as the approximate date for onset of symptoms. Red crosses represent individuals lost to follow up. (For interpretation of the references to colour in this figure legend, the reader is referred to the web version of this article.)

Symptoms were long-lasting but resolved gradually (Fig. 3).

A total of 637 participants (86%) (374 girls, 263 boys) reported a full recovery from their symptoms at their last contact; the subcutaneous nodules at the injection site had disappeared and they had not been itching for at least one year. The median duration of itching in the recovered group was 6.6 years (range 0.04–19.2 years). Another 108 adolescents (81 girls, 27 boys) reported ongoing symptoms for 0.1–25.2 years (median 16.4 years) in their last interview/questionnaire. All but one graded their remaining symptoms as “Improved” or “Nearly recovered”. Among the 108 individuals who reported itching at the last contact, 30 (28%) could not be reached after 2008 despite several phone and postal service attempts. Many of them had moved abroad.

In the physical examination that preceded the patch test of 241 children in 2007–2008, remaining nodules were found in 5 children, local discolouration in 26, hypertrichosis in 5 and visible excoriations in 8 children.

### Observations after DT-booster vaccination

Among the 745 children in the study, 430 received a DT-booster dose at about 10 years of age within the Gothenburg Pertussis Vaccine Trial as long as the aluminium-free and aluminium phosphate vaccines were available. Another 293 children were vaccinated in the School Health Care with commercial aluminium hydroxide adsorbed DT vaccines.

Of the altogether 723 children who received a DT-booster, 23 (3.2 %) reported new itching and/or local swelling or subcutaneous nodules at the injection site. All had received an aluminium adjuvant vaccine, mostly Duplex® (Table 3). No participants presented with symptoms for more than some weeks to some months.

**Table 3:** Reported new itching and/or local swelling on the injection site after DT-booster vaccination in participants with itching nodules after vaccination in the Gothenburg Pertussis Vaccine Trials.

	*Type of diphtheria / tetanus(DT)-booster vaccine*			
	Al-free	Duplex®	Commercial*	Other**
Total number of participants	115	315	293	22
Itching at new injection site after DT-booster	0 (0%)	17 (6%)	6 (2%)	–

*Mainly diTeBooster ®. If this was not available, a DTaP combination vaccine was given.

**DT-booster vaccination received earlier (n = 11), denied (n = 7) or unknown (n = 4).

### Reported symptoms after other aluminium containing vaccinations and allergen-specific immunotherapy

In all, 332 individuals reported further vaccinations with other aluminium adjuvant vaccines (hepatitis A and B, HPV, TBE).

Of them, 24 individuals reported itching on the new vaccination site for some months. Two also presented with subcutaneous nodules. Another 3 participants were treated with repeated injections of aluminium-adsorbed allergen extracts; all developed new itching nodules. Their treatment required discontinuation or replacement with aluminium-free ASIT. The duration of these symptoms is unknown.

As many as 326 children/adolescents reported that they had abstained from further vaccinations with aluminium-adsorbed vaccines. The vaccination history for the remaining 84 individuals is unknown.

## Discussion

To the best of our knowledge, no other long-term follow-up of clinical prognosis of vaccine-related itching nodules has been reported in a large study population. In the present study, 745 children with persistent itching subcutaneous nodules at the injection site for aluminium-adsorbed aP vaccines could be followed prospectively for more than 20 years (1997–2019).

### The clinical course

A prolonged course is well known and typical for aluminium-induced itching granulomas, described by several authors, though mostly in general terms (“continued for months or years”) and mainly as case reports [6], [20], [21], [22], [23]. In a Danish study, 21 children with vaccination granulomas were followed for 1–8 years; 5 recovered, 11 improved, and 5 remained unchanged [5]. In a Swedish study from 2005 [24], 19 cases of persistent pruritic nodules were described after commonly used aluminium-adsorbed vaccines and followed for 1–7 years (median 4.5 years). At the latest contact, two were recovered after 2.5–3.5 years of symptom duration, 12 were improved but 5 still experienced intense itching for the last 1 to 2 years.

In the present study the median duration of symptoms was 6.6 years for the 86% of the individuals who were fully recovered. The remaining participants reported ongoing symptoms when they were last interviewed, but all except one graded their symptoms as “Improved” or “Nearly recovered”. An even higher recovery rate than 86% is likely, as more than a quarter of the whole group has been lost to follow up since 2008. The result is consistent with another Swedish study of 64 spontaneously reported children with itching nodules following vaccination [25]. Here the median duration of symptoms was 4.6 years for the 37% who had recovered. The remaining children still had mild to severe itching after 6 years. In a Danish report on 38 children, 84% experienced persistent itching with a median duration of 1.6 years at the time of the study [26]. However, their data are hard to compare with our results as they had no ongoing long term follow up.

As earlier described, the experienced symptoms gradually resolved with time. The nodules disappeared before the pruritus. The scratching, initially sometimes until skin bleeding, decreased and became intermittent and less intense. In the last symptom phase, “Nearly recovered”, long periods (months) free from symptoms were interrupted by mild itching for a while, often triggered by an upper respiratory tract infection. The skin manifestations in the itching area were normalised.

### Future vaccinations with aluminium-adsorbed vaccines

A main finding in this study is that only 3% of the participants who were DT-booster vaccinated and 7% of those who reported vaccination with other aluminium-adsorbed vaccines later on in life experienced new itching at the injection site, but only for a short period. A few similar reactions were also described in other studies [25], [26]. Unexpectedly, as there had been no earlier reports, mainly those children vaccinated with an aluminium phosphate DT-booster (Duplex®) reported new itching.

Three individuals in our study who later in life were treated with ASIT all presented with new itching nodules at the injection sites for the allergen extracts, one of them with ongoing symptoms. Itching aluminium granulomas appearing during treatment with ASIT is a well known adverse event reported by several authors [4], [5], [23], [27]. Children with vaccine-induced itching nodules should be informed of this risk so that aluminium-free extracts could be used in case ASIT would be needed later on in life.

An unfortunate consequence when “bumps” as large as up to 15–20 mm suddenly appeared under the skin in a child was that they were mistaken for tumours. At least 5 children in the Gothenburg trials were investigated for malignancy with ultrasound, biopsy or extirpation in general anaesthesia [19]. Such investigations are also described in other studies [22], [25], [28].

A risk for new problems after future vaccinations with aluminium-adsorbed vaccines has been anticipated by many authors. The general recommendation in the 1990 s was to avoid such vaccinations [6], [20], [23], [29], [30]. Also parents feared further vaccinations. The DTaP vaccination series were interrupted for 35 infants in our study when itching nodules appeared after the first or second dose [19], and the DT-booster dose at 10 years was omitted for 7 children. Postponed or denied DT-booster vaccinations are also described in other studies [25], [26]. As many as 55% of the adolescents in our study had refrained from important vaccinations later on in life (Hepatitis A and B, HPV and TBE) due to the aluminium content of the vaccines.

At the beginning of our study, it was difficult to give good advice and recommendations concerning future vaccination. In the Gothenburg Mass Vaccination Project (1995–99), 3 doses of the study vaccines were given in the left thigh or upper arm at 2- and 6 month intervals. Itching nodules appeared mostly after the third dose but 4% of the children had onset already after the first dose and 18% after the second dose. During the first years of the trial, the following dose(s) were given in another limb, leading to itching nodules in 2 sites for 75 children and in 3 sites for 4 children.

Thus the risk for new persistent itching nodules on the site for the following doses was substantial when they were given at a short interval (2–6 months) from the one that caused the original itching nodule. On the other hand, children who received the DT-booster with commercial vaccines with a long interval (many years) after the original itching nodule very seldom experienced a new itching nodule. The risk clearly decreases with time. Our present recommendation for children with itching nodules after vaccination with aluminium-adsorbed vaccines in infancy is, therefore, that future vaccinations with aluminium-adsorbed vaccines can be performed with little risk for new itching nodules when the child has completely recovered.

Infants with the onset of itching nodules already after the first or second dose of DTaP and/or pneumococcal vaccine at 3 and 6 months of age may have an enhanced risk for new reactions after the following dose(s) [1], [7], [25]. They should be judged individually and the intensity of symptoms must be weighed against the consequences of a postponed vaccination series.

### Limitations

In our study, the approximate duration of itching was fairly well known in most individuals due to repeated contacts for many years. However, there is, of course, some uncertainty, especially in those with less contact. It is easier to estimate when something began than when it ceased.

The compliance with the childhood vaccination program in Sweden is very high [31], and the School Health Care system in the area was regularly informed, but still, there is some uncertainty about the history of the DT-booster vaccination. Another limitation is data concerning additional vaccination with other aluminium-adsorbed vaccines since the young participants were not always aware of their vaccination history.

A possible source of bias could be that all participants were participating in a clinical trial. Participants in clinical trials may be more keen in reporting clinical side effects. The same may be true for the trial staff, who are more likely to look for side effects.

During the present time, with much hesitation among parents to vaccines because of side effects supported by large-voiced vaccine opponents in non-scientific social media, it may be challenging to write about side effects of vaccines. Therefore, it must be stressed that vaccines are one of to the most important developments to prevent serious diseases and have prevented millions of deaths in both children and adults. In our opinion, it is important to recognise, study and report the side effects of vaccines, not only to improve future vaccines but also so that vaccine opponents cannot use the argument that side effects are concealed by authorities. The personal opinion of all authors of the present study is that vaccinations are essential for public health, even though it cannot be denied that side effects can occur.

## Conclusion

This study shows that vaccine-induced itching granulomas caused during childhood by an aluminium-adsorbed aP vaccine used in Gothenburg’s clinical trials in the 1990 s seem to disappear over time and lack clinical significance when the participants were re-exposed to other aluminium-containing vaccines.

We recommend that further vaccination with aluminium-adsorbed vaccines can continue for older children once the original nodule has vanished and the itching has resolved or nearly resolved. Infants with the onset of symptoms already after the first or second dose should be judged individually. Alternatives to aluminium adjuvants should be considered.

## Declaration of Competing Interest

The authors declare that they have no known competing financial interests or personal relationships that could have appeared to influence the work reported in this paper.
